# A Novel Epigenetic Regulator ZRF1: Insight into Its Functions in Plants

**DOI:** 10.3390/genes12081245

**Published:** 2021-08-15

**Authors:** Jing Feng, Yahui Gao, Kun Wang, Mingguo Jiang

**Affiliations:** Guangxi Key Laboratory for Polysaccharide Materials and Modifications, School of Marine Sciences and Biotechnology, Guangxi University for Nationalities, Nanning 530008, China; fengjing0414141@163.com (J.F.); gyh082516@163.com (Y.G.); wudishuaikun@163.com (K.W.)

**Keywords:** ZRF1, *Arabidopsis thaliana*, stem cell maintenance, embryo development, monoubiquitination

## Abstract

Recently, Zuotin-related factor 1 (ZRF1), an epigenetic regulator, was found to be involved in transcriptional regulation. In animals and humans, ZRF1 specifically binds to monoubiquitinated histone H2A through a ubiquitin-binding domain and derepresses Polycomb target genes at the beginning of cellular differentiation. In addition, ZRF1 can work as a tumor suppressor. According to bioinformatics analysis, ZRF1 homologs are widely found in plants. However, the current studies on ZRF1 in higher plants are limited and few in-depth studies of its functions have been reported. In this review, we aim to summarize the key role of AtZRF1a/b in *Arabidopsis thaliana* growth and development, as well as the research progress in this field in recent years.

## 1. Introduction

Epigenetics is the study of inherited changes in phenotype (appearance) or gene expression caused by mechanisms other than the changes in the underlying DNA sequence; hence, the name epi- (Greek: επί- over, above) genetics. Epigenetic regulatory mechanisms comprise histone post-translational modifications, histone variants, DNA methylation, and ATP-dependent chromatin remodeling, which are essential for controlling patterns of gene expression during the cell cycle, development, and in response to environmental stimuli. More importantly, epigenetic changes may be reversed [1,2]. DNA methylation has been generally considered a heritable chromatin modification mark that plays important roles in maintaining chromosome stability, regulating transposon silencing, and regulating gene expression [3,4]. Histone post-translational modifications include methylation, acetylation, phosphorylation, ubiquitination, and sumoylation; they are dynamic changes and play key roles in regulating chromatin structure and gene expression, mainly by changing chromatin compaction and shaping the three-dimensional topology of the genome [5,6,7].

Polycomb Group (PcG) proteins are evolutionarily conserved transcriptional regulators, which are essential for the control of development and cell differentiation in plants and animals. Polycomb-mediated transcriptional regulation is mediated by two major multisubunit complexes: Polycomb Repressive Complex 2, which catalyzes histone H3 lysine 27 trimethylation (H3K27me3) on target chromatin, and PRC1, which not only recognizes and binds H3K27me3, but also catalyzes the mono-ubiquitination of histone H2A lysine 119 (H2AK119ub1), resulting in a stable silencing chromatin state [8].

Zuotin-related factor 1 (ZRF1) (also known as DNAJC2, MIDA1, Mpp11, and GlsA) has been characterized as an epigenetic factor involved in transcriptional regulation. In most species, ZRF1 protein is evolutionarily conserved. The N-terminal of all homologs of ZRF1 has a zuotin domain, which is composed of a DnaJ domain and a potential ubiquitin-binding domain (UBD). The C-terminal contains tandem repeats of SANT (Swi3, Ada2, NcoR1 and TFIIIB) domain, but only exists in higher eukaryotes [9,10]. It has been reported that human ZRF1 protein can specifically recognizes and bind to monoubiquitinated histone H2A through UBD, and activates polycomb-repressed target genes through the E3 ubiquitin ligase activity of Ring1B at the onset of cellular differentiation (e.g., stem cell differentiation, the neuronal lineage, and mesodermal lineage) [10,11,12,13]. In mice, based on the CRISPR/Cas9 approach, it was first suggested that DNAJC2 was necessary during early mouse embryogenesis [12]. Moreover, the nuclear epigenetic factor ZRF1 participates in senescence and is involved in certain cancers [14,15,16,17]. Research evidence suggests that ZRF1 can be phosphorylated on Ser47 residues by S6 kinases 1 and 2 (S6Ks) and participates in a senescence programme by regulating the cell cycle inhibitor p16 [18]. It is very interesting that ZRF1 has a dual role. Not only does it work as a tumor suppressor, but it can also induce carcinogenesis depending on the cellular context [14,19]. In addition, ZRF1, a switch protein, remodels chromatin-bound E3 ligases during lesion recognition [20]. Under ultraviolet radiation, the endonuclease DICER and ZRF1 are required for chromatin decondensation. DICER was recruited into chromatin in a ZRF1-mediated manner. H2A ubiquitin-binding protein ZRF1 interacts with DICER to affect chromatin conformation through chromatin remodeling factors PARP1 [21].

Over the past several years, the functions of ZRF1 in animals and humans have been identified and have become a new research hotspot. In most species, ZRF1 is evolutionarily conserved [9,22]. According to bioinformatics analysis, ZRF1 homologs are widely found in plants (e.g., *A. thaliana*, *Lilium longiflorum*, *Alstroemeria aurea*, *Volvox carteri*), and *LlglsA* (*L. longiflorum gonidialess A*) was identified as the first higher plant homolog of *glsA*/ZRF1 [22,23,24,25]. However, current studies of ZRF1 in plants are limited, and few in-depth studies of its functions have been reported. In this review, we aim to summarize recent advances in the functions of ZRF1 in *A. thaliana* with a focus on recent progress in the field.

## 2. ZRF1 Orthologs and the Domain Structure in *Arabidopsis*

In *Arabidopsis*, two ZRF1 proteins (AtZRF1a and AtZRF1b) have been identified, and they share 81% sequence identity. *AtZRF1a* gene, encoding a 74 kDa protein consisting of 647 amino acids, is located on chromosome 3 (At3g11450). *AtZRF1b* gene, encoding a 75.6 kDa protein composed of 663 amino acids, is located on chromosome 5 (At5g06110). There are no introns within the coding region of both *AtZRF1a* and AtZRF1b [22]. Both *AtZRF1a* and *AtZRF1b* are broadly expressed in various plant organs/tissues, and the single loss-of-function mutants *atzrf1a* and *atzrf1b* showed normal phenotypes, while the double mutants *atzrf1a atzrf1b* have a pleiotropic phenotype [22]. Taken together, the data demonstrate that *AtZRF1a* and *AtZRF1b* have redundant functions [22].

**Figure 1:**
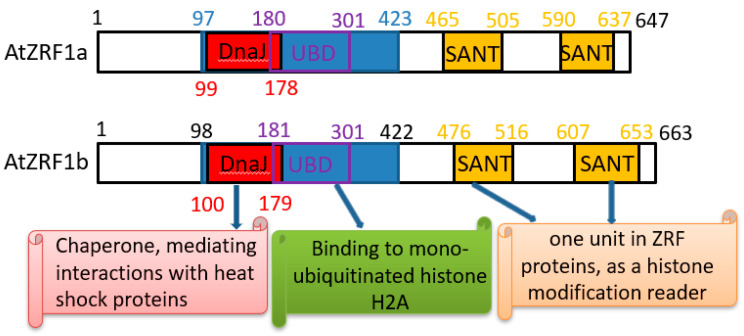
Schematic diagram of *Arabidopsis* ZRF1a/b for conserved functional domain organization.

**Figure 2:**
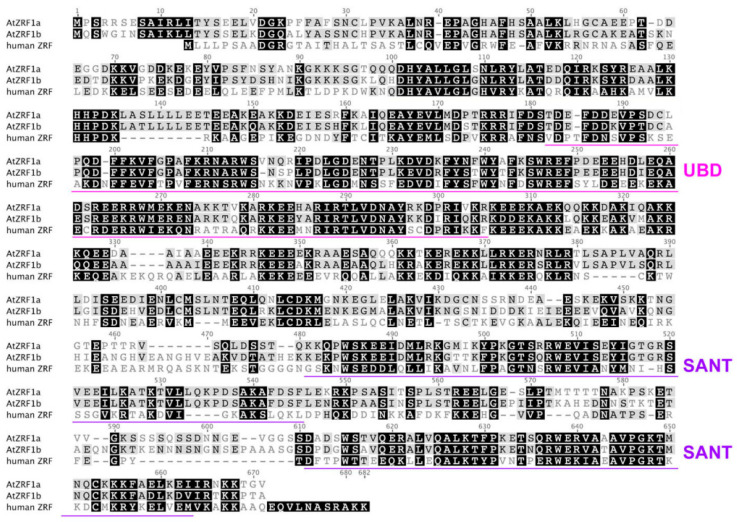
Sequence alignment of AtZRF1a and AtZRF1b together with the human ZRF1. The alignment was generated using the CLUSTALW program. The UBD and SANT domains are indicated [22].

The ZRF1 protein structure is highly evolutionarily conserved, and several domains have been annotated [9,14]. Similar to human ZRF1, AtZRF1a and AtZRF1b also contain two major recognizable types of structural domains, namely, the Zuotin domain (Z-DNA binding domain) and SANT (Swi3, Ada2, NcoR1, and TFIIIB) domains (Figure 1 and Figure 2). Zuotin contains a highly conserved DnaJ domain (J-domain), which is similar to the mammalian HSP-40 (heat shock protein 40) chaperone, mediating interactions with heat shock proteins (Hsp70). The green alga *V. carteri* ZUO1/ZRF ortholog Gonidialess A (GlsA) can bind to the corresponding Hsp70 chaperones, and this interaction is required for asymmetric cell division [26,27]. Zuotin also contains a ubiquitin-binding domain (UBD) domain/M region, which is adjacent to the C terminus of the J-domain. Human MPP11/ZRF1 can bind to the H2Aub epigenetic marker and replace Polycomb repressive complex 1 (PRC1) through the UBD domain [10]. Interestingly, AtZRF1b can bind ubiquitin and can pulldown H2Aub1 via the UBD domain; the loss of AtZRF1a/b does not cause an increase in H2Aub1 but rather results in a very slightly reduced global level of H2Aub1 in *atzrf1a-1 atzrf1b-1* mutant plants [22]. Unfortunately, there is no relevant experimental evidence for the interaction between ZRF1a and H2Aub. Except for fungal proteins, all known ZRF1 orthologs contain the tandem repeat of SANT domains at the C terminus. SANT domain proteins can recruit histone acetylases or deacetylases to modify histone tails [28,29,30], but their specific functions remain to be addressed in plants.

## 3. ZRF1 Is Required in Seed/Embryo Development in *Arabidopsis*

A seed consists of a plant embryo and an endosperm enclosed by a seed coat. Several studies have shown that PRC1 is involved in seed development and seedling growth [31,32,33]. The double mutants *Atring1a Atring1b*, as well as *Atbmi1a Atbmi1b*, exhibit derepression of embryonic traits in somatic plant tissues, due to the lack of AtRING1 or AtBMI1, the key catalytic factors of PRC1. For example, ectopic callus formed in cotyledon, leaf or SAM region of mutant plants [31,32]. CURLY LEAF (CLF), EMBRYONIC FLOWER2 (EMF2), VERNALIZATION2 (VRN2) and SWINGER (SWN) are PRC2 components, the *clf swn* and *emf2 vrn2* mutants also exhibit ectopic cell dedifferentiation and somatic embryogenesis [34]. Investigation of the triple mutants *Atring1a Atring1b clf* revealed that the expression of embryo regulatory genes was increased [32,35]. These genetic data suggest that PRC1 and PRC2 jointly regulate embryonic regulatory genes to prevent somatic dedifferentiation.

Compared with wild-type seeds, the seeds of *atglsa1-3 atglsa2-1* double mutants are shriveled, wrinkled, distorted, and small [23]. In *atzrf1a-1 atzrf1b-1* and *atzrf1a-2 atzrf1b-1* double mutants, plants are poorly fertile and seed abortion is observed. Based on reciprocal crosses of heterozygous mutant plants with wild-type Col-0 plants, male and female transmissions were impaired. Some seedlings (about 35%) exhibited a single cotyledon, asymmetrical cotyledons, and fleshy narrow cotyledonous phenotypes, which display some defects similar to *Atbmi1aAtbmi1b* and *Atring1aAtring1b* [22]. Through electron microscopy, severe defects in all stages of embryo development were observed, such as at the pre-globular, octant stage, unorganized division, and a lack of suspensor development; at the globular stage, failure of hypophysis development [23]. These phenomena suggest that ZRF1a/b plays a very important role in embryo development. In humans/mice, PRC1 and PRC2 are epigenetic repressors required for proper embryonic stem cells differentiation and embryo development [36]. ZRF1 can replace PRC1 from Chromatin by competing for H2Aub1 markers, thus inducing de-repression of neural genes [10,11]. However, in plants, the relationship between ZRF1 and PRC1/PRC2 in regulating embryonic development is not clear.

Seed germination is the first step in plant postembryonic growth, which is regulated by various pathways. The primary energy for seed germination in higher plants comes from seed storage products. The expression of seed developmental genes (e.g., *ABI3* (*ABA INSENSI-TIVE 3*), *CRA1* (*CRUCIFERIN1*), *CHO1* (*CHOTTO1*)) should be silenced from seed germination to the whole plant growth and development process [37,38,39]. Under standard growth conditions, the double mutants *atzrf1a-1 atzrf1b-1* and *atzrf1a-2 atzrf1b-1* showed delayed seed germination. *ABI3*, *CRU1/CRA1*, *CRU3*, and *CHO1/AIL5*, were up-regulated in *atzrf1a atzrf1b* mutants as well as in *atbmi1a atbmi1b* mutant seedlings. Further, within the *ABI3*, *CRU3*, and *AIL5* gene loci, both H2Aub1 and H3K27me3 levels were strongly reduced. Previous studies have shown that de-suppression of *ABI3*, *CRU3*, and *AIL5* expression is associated with decreased levels of H2Aub1 and H3K27me3 in *atbmi1a atbmi1b* mutants [22,33]. These results indicate that ZRF1a/b is required to enhance H2Aub1 and H3K27me3 levels for suppressing seed developmental genes in seedlings, similar to AtBMI1a/b. Since ZRF1 is associated with H2Aub1 de-deposition in animals, it is suspected that the complementary mechanisms of the interaction between AtZRF1A/B-H2Aub1 and AtZRF1A/B-PRC1/PRC2 may be involved in the deposition of H2Aub1 and H3k27me3, thus effectively silencing seed development genes and promoting plant vegetative growth. However, further experiments are needed to test this hypothesis.

## 4. ZRF1 Maintains the Function of Stem Cells in *Arabidopsis*

Plant growth and development are largely dependent on stem cells located in the shoot apical meristem (SAM) and root apical meristem (RAM), whose activities are fine-tuned by multi-family chromatin factors [40,41]. For plants, the transition from vegetative growth to reproductive growth is a critical process that is caused by the cell fate transformation of SAM into the inflorescence meristem (IM). IM produces primary and secondary flowering stems and subsequently the formation of flowers.

## 5. ZRF1 Affects Shoot Apical Meristem Activity in *Arabidopsis*

The double mutants *atzrf1a atzrf1b* exhibited ectopic SAM initiation and fasciated stem [22]. The apical meristem dominance was absent in *atglsa1-3 atglsa2-1* double mutants, and near SAM, new meristems were ectopically initiated [23]. The balance between stem cell maintenance and organ-forming cell differentiation is controlled by specific transcription factors, including *KNOX* (*Class I KNOTTED1-like homeobox*) proteins. Strikingly, several *KNOX* genes, e.g., *STM* (*SHOOT-MERISTEMLESS*), *BP* (*BREVIPEDICELLUS*)/*KNAT1*, *KNAT2*, and *KNAT6*, were up-regulated in *atzrf1a atzrf1b* double mutants. Moreover, based on *STM-GUS* expression pattern, there were twin rather than single SAMs in *atzrf1a atzrf1b* double mutants [22,23]. These are similar to that of the previously reported *atring1a atring1b* and *atbmi1a atbmi1b* mutants [32,35], the findings indicate that AtZRF1a and AtZRF1b play redundant roles in stably inhibiting stem cell activity to allow appropriate lateral organ differentiation, and AtRING1A/B, AtBMI1A/B and AtZRF1A/B may act on a common set of genes and maintain normal SAM activity during plant development in Arabidopsis.

## 6. ZRF1 Is Essential for Organization and Maintenance of the Root Stem Cell Niche in *Arabidopsis*

In *atzrf1a atzrf1b* double mutants, the length of primary roots was only 1/3 that of the wild type, and many adventitious roots were produced [23,42]. The size of RAM in mutants was smaller than that in the wild type. Through root cell-type-specific markers introgressed into the *atzrf1a atzrf1b* double mutants, it was found that green fluorescent protein (GFP) driven by the promoter of *WUSCHEL-RELATED HOMEOBOX 5* (*WOX5*::*erGFP*) was expressed specifically in the quiescent center (QC) cells in wildtype, however, in the *atzrf1a atzrf1b* mutant, QC cells showed abnormal morphology, *WOX5*::*erGFP* was expressed not only in QC cells, but also in adjacent cortical/endothelial cells. The expressions of the *J2341* enhancer trap marker and *SCARECROW* (*SCR*)::*SCR-YFP* endodermis marker were found to be decreased in *atzrf1a atzrf1b* mutants. Moreover, in mutants, the cortex layer showed low or no CO2::*H2B-YFP* expression. All these results indicated that AtZRF1a/b plays a crucial role in the establishment and maintenance of cell fate of various cell types in RAM [42]. The *atzrf1a atzrf1b* double mutants showed increased expression of several key root regulatory genes, including *WOX5* and *ERF115* (associated with dividing quiescent center cells) [43]. Taken together, these findings indicated that AtZRF1a/b was involved in the regulation of root development.

## 7. ZRF1 Is Involved in Flower Development in *Arabidopsis*

Flowers are the sexual reproductive structures of flowering plants or angiosperms. A wild-type Arabidopsis floret consists of a fixed number of floral organs, including four sepals, four petals, six stamens, and two fused carpels. In contrast, the number of these organs is decreased in some *atzrf1a atzrf1b* double mutant flowers (e.g., fewer sepals, fewer petals, fewer stamens) [22]. In addition, *atzrf1a atzrf1b* double mutants had fasciated primary inflorescence stems and abnormal secondary flowering stems (e.g., a flower forming inside of a flower) [22]. All these phenotypes indicate that AtZRF1a/b is involved in the correct differentiation of inflorescence stem cells. At present, we only know that the expression of the organic-boundary gene *CUP SHAPED COTYLEDON 1* (*CUC1*), *CUC2* and *CUC3* were up-regulated and the expression of floral homeotic gene *AGAMOUS* was down-regulated in zrf1 double mutants. Unfortunately, no studies have been conducted on the mechanism of its regulation of floral organ development.

## 8. Other Functions of ZRF1 in *Arabidopsis*

It is well known that plant hormones are important in root development. The *atzrf1a atzrf1b* double mutants were observed to be involved in abnormal root development [23,42]. In the determination of plant growth sensitivity to auxin treatment, it was found that the *atzrf1a atzrf1b* double mutants were more sensitive than the wild type, based on the addition of exogenous1-naphthlcetic acid. Furthermore, the auxin response reporter *DR5rev::GFP* was introgressed into the *atzrf1a atzrf1b* double mutants, and it was found that the loss of function of ZRF1a/b had a negative effect on auxin distribution [42]. The cytokinin-response reporter *TCS::GFP* was introgressed into the *atglsa1-3 atglsa2-1* double mutants, and the results showed that the localization of cytokinin changed in mutants [23].

Analysis of ploidy levels and the expression levels of cell cycle genes (e.g., *CYCD4;1*, *KRP6*) in *atzrf1a atzrf1b* double mutants true leaves showed that AtZRF1a/b inhibited the transition of mitosis to the endocycle [22]. Moreover, the mutant roots displayed low mitotic activities and elevated polyploid levels [42]. These results indicated that AtZRF1a/b is involved in the regulation of the cell cycle programme.

## 9. The Function of zrf1 in Other Plants

In *V. carteri*, GlsA has been reported as a chaperone-like protein, which is required for germ cell specification [27]. The *glsA* mutants in the green alga *V. carteri* had a tight gonidialess (Gls) phenotype, based on CRISPR/Cas9, demonstrating that *GlsA* is essential for asymmetric cell division [44]. Hsp70A and GlsA interact as partner chaperones to regulate asymmetric division [26]. The *glsA* mutant of green alga *V. carteri* showed hypersensitivity to both cold and the ribosome-binding aminoglycoside antibiotic paromomycin, suggesting that GlsA probably regulates translational accuracy [45].

In *L. longiflorum*, the *LlglsA* gene accumulated preferentially in the generative cell during pollen development and was not strongly expressed during the asymmetric division stage and in various somatic cells. LlglsA interacts with microtubule filaments under specific conditions (e.g., bending, polymerizing), which plays an important role in the morphogenesis of the generative cell [25]. In addition, a functional GlsA ortholog of higher plants was isolated from *A. aurea*. Based on expression analysis, *AaglsA* plays roles in both somatic and reproductive tissues in *Alstroemeria* [24]. These results indicate that ZRF1 has plant-specific features, but the detailed mechanisms underlying the reproductive process still have to be elucidated.

## 10. Conclusions and Perspectives

The ZRF1 proteins in eukaryotes play a crucial role in several processes, including stem cell maintenance, cell-fate decisions, differentiation, senescence, and cancer. In Arabidopsis, two ZRF1 homologs, AtZRF1a and AtZRF1b, have redundant functions, and they are widely expressed in almost all young organs of plants [22,23]. They are key regulators of multiple processes related to dividing cells and meristematic tissues during plant growth and development [22,23]. However, the role of ZRF1 in plants remains unclear.

In the working model of human ZRF1, as a chromatin-binding protein, ZRF1 recognizes and binds to monoubiquitinated histone H2A (H2Aub1); then, ZRF1 can replace PRC1 from chromatin by competing for the H2Aub1 mark [10]. It is indicated that (1) ZRF1 as a transcriptional activator is due to the removal of the H2Aub1 mark; (2) ZRF1 plays a role in a polycomb-dependent manner. However, increasing evidence suggests that ZRF1 might interact with the facilitated chromatin transaction complex and/or the mixed-lineage leukemia complex to participate in transcriptional activation [14]. These processes are not due to the removal of the H2Aub1 mark. In acute myeloid leukemia cells, ZRF1 works as a transcriptional repressor [19], and mechanistic studies have shown the dual role of ZRF1, as it can function either as a tumor suppressor or induce carcinogenesis depending on the cellular context [14,19]. Polycomb proteins have a function independent of ZRF1 in neurogenesis [14]. ZRF1 functions might depend on the relationship of distinct complexes. Indeed, many protein factors have been identified to be associated with ZRF1 (Table 1). For example, inhibitor of differentiation 1 (ID1) physically interacts with ZRF1 and has been shown to impair ZRF1 binding to chromatin, thus preventing the activation of neural genes in embryonic stem cells [14]. In myeloid leukemic cells, the RA receptor α (RARα) interacts with ZRF1 to regulate RA target gene transcription [19]. Additionally, in ESCs and differentiating cells, ZRF1 interacts with CDK8 to recruit CDK8 to promoters of its target genes, and ZRF1 mediates the interaction of Cdk8 with Med12 [46]. Med12, a member of the large mediator complex, has a critical and central role in RNA polymerase II transcription. During differentiation, ZRF1 acts as a switch protein that remodels mediator-associated protein complexes, thereby converting repressive Mediator (Med12-Ring1b) into an active enhancer (Med12-Cdk8) [46].

The loss of function of AtZRF1a/b affects the expression of a large number (4519) of genes, approximately half of which are up-regulated and half of which are down-regulated [22]. Gene Ontology (GO) analysis revealed that the differentially expressed genes had a fairly similar distribution among *atzrf1a-1 atzrf1b-1* and the PRC1-deficient mutants including *atbmi1a atbmi1b* and *atring1a atring1b* mutants. However, only 114 genes were up-regulated in all three mutants, the up-regulated genes are highly expressed in the process of seed development, which is consistent with the key roles of AtZRF1A/B, AtBMI1A/B and AtRING1a/b in seed germination and seedling development. Seventy-five percent of these commonly up-regulated loci are marked by H3K27me3, which is consistent with their silencing via polycomb mediated suppression. A large number of misregulated genes were found specifically in *atzrf1a-1 atzrf1b-1* but not in *atbmi1a atbmi1b* and/or *atring1a atring1b*. The evidence suggests that the function of ZRF1 in regulating the homeostasis of H2Aub1 may be context-dependent. AtZRF1a/b have both PRC1-related and independent functions in regulating plant growth and development [22]. Loss of ZRF1 function affects the expression of a large number of genes, and AtZRF1A/B may be involved in the removal of H2Aub1 from some genes in Arabidopsis, and vice versa, and may be involved in the deposition of H2Aub1 from some genes that have not yet been detected. Current studies on the function of ZRF1 in plants are very limited, and it is not clear which transcription factors or regulatory factors can interact with ZRF1; thus, we cannot speculate on the mechanism of ZRF1. Therefore, in the future, it is necessary to further study the role of ZRF1 under different developmental conditions and to reveal the mechanism underlying the role of ZRF1 in plant growth and development.

## Figures and Tables

**Table 1:** List of protein factors reported as associated together with ZRF1.

	Associated Factor	Function	Interaction Assay	Reference
ZRF1	Id1 Gene ID: 3397	the protein inhibitor of DNA binding	Pulldown, CoIP	[10,14]
	RARα Gene ID: 5914	the RA receptor α	Pulldown, CoIP	[19]
	DICER Gene ID: 23405	endoribonuclease	CoIP, IP	[21]
	XPC Gene ID: 7508	DNA lesion recognition factor	IP	[20]
	CDK8 Gene ID: 1024	cyclin-dependent kinase 8	IP	[46]
	H2Aub1	monoubiquitinated histone H2A	Pulldown	[10]
	HSP70L1 Gene ID: 51182	Heat shock protein 70-like protein	CoIP	[47]
	Hsp70A Gene ID: 5726860	Heat shock protein 70	CoIP	[26]

CoIP: co-immunoprecipitation; IP: immunoprecipitations.

## Data Availability

The study did not report any data.
